# Composition and Anticoagulant Potential of Chondroitin Sulfate and Dermatan Sulfate from Inedible Parts of Garfish (*Belone belone*)

**DOI:** 10.3390/foods12213887

**Published:** 2023-10-24

**Authors:** Sawssen Ben Chikha, Hajer Bougatef, Federica Capitani, Ikram Ben Amor, Francesca Maccari, Jalel Gargouri, Assaad Sila, Nicola Volpi, Ali Bougatef

**Affiliations:** 1 Laboratory for the Improvement of Plants and Valorization of Agroressources, National School of Engineering of Sfax (ENIS), University of Sfax, Sfax 3038, Tunisia; benchikhasawsen570@gmail.com (S.B.C.); hajer.bougatf93@gmail.com (H.B.); assaadsila@gmail.com (A.S.); 2 Clinical and Experimental Medicine Ph.D. Program, University of Modena and Reggio Emilia, 41121 Modena, Italy; federica.capitani@fmail.com; 3 Sfax Regional Blood Transfusion Center, El-Ain Road km 0.5, Sfax 3003, Tunisia; ikrambenamor.hemato@gmail.com; 4 Department of Life Sciences, University of Modena and Reggio Emilia, Via Campi 213/D, 41125 Modena, Italy; francesca.maccari@unimore.it (F.M.); nicola.volpi@unimore.it (N.V.); 5 Laboratory of Hematology, Medical Faculty of Sfax, University of Sfax, Magida Boulila Avenue, Sfax 3029, Tunisia; jalelgargouri@yahoo.fr; 6 Department of Life Sciences, Faculty of Sciences of Gafsa, University of Gafsa, Gafsa 2100, Tunisia; 7 High Institute of Biotechnology of Sfax, University of Sfax, Sfax 3038, Tunisia

**Keywords:** *Belone belone*, by-products, chondroitine sulfate, dermatan sulfate, chemical characterization, anticoagulant activity

## Abstract

Glycosaminoglycans (GAGs) play a crucial role due to their significant biomedical functions. Chondroitin sulfate (CS) and dermatan sulfate (DS), the main representative family of GAGs, were extracted and purified from garfish (*Belone belone*) by-products, i.e., skin (GSB), bones (GCB), and heads (GHB), and their composition and anticoagulant activity were investigated. CS/DS were purified by ion-exchange chromatography with yields of 8.1% for heads, 3.7% for skin, and 1.4% for bones. Cellulose acetate electrophoresis was also explored for analyzing the extracted CS/DS. Interestingly, GHB, GSB, and GCB possessed sulfate contents of 21 ± 2%, 20 ± 1%, and 20 ± 1.5%, respectively. Physico-chemical analysis showed that there were no significant differences (*p* > 0.05) between the variances for sulfate, uronic acid, and total sugars in the GAGs extracted from the different parts of fish. Disaccharide analysis by SAX-HPLC showed that the GSB and GCB were predominately composed of ΔDi-4S [ΔUA-GalNAc 6S] (74.78% and 69.22%, respectively) and ΔDi-2,4S [ΔUA2S-GalNAc 4S] (10.92% and 6.55%, respectively). However, the GHB consisted of 25.55% ΔDi-6S [ΔUA-GalNAc 6S] and 6.28% ΔDi-2,6S [ΔUA2S-GalNAc 4S]. Moreover, classical anticoagulation tests were also used to measure their anticoagulant properties in vitro, which included the activated partial thromboplastin time, prothrombin time, and thrombin time. The CS/DS isolated from garfish by-products exhibited potent anticoagulant effects. The purified CS/DS showed exceptional anticoagulant properties according to this research and can be considered as a new agent with anticoagulant properties.

## 1. Introduction

Diseases of the blood circulation system are a real public health problem that can cause mortality, especially in developed nations [1]. The coagulation pathway of the blood circulation system is inhibited by enzymes. Currently, marketed heparins are mainly extracted from the intestinal mucosa of pigs or from bovine lung and intestinal tissues [2]. These drugs offer a rapid and effective anticoagulant effect, the mode of action of which is mainly due to their binding to antithrombin (AT). Despite their effectiveness in treating thrombotic pathologies, the excessive use of these molecules has a number of disadvantages, namely hemorrhagic complications and thrombocytopenia [3]. Moreover, animal heparins can contain pathogen agents like viruses or prions, which can be responsible for health risks. Fish-derived heparins have been considered as a safer source because they are less likely to carry known infectious agents like prions and certain viruses. Additionally, the fish used for heparin extraction are typically sourced from regulated and monitored fisheries, reducing the likelihood of contamination. On the other hand, the low bioavailability of heparin makes it a very expensive treatment. Thus, given the risks and costs of these treatments, there is a real need to find new sources of anticoagulants. The limitations of conventional heparin treatment have motivated researchers to find new heparin-like anticoagulants acting at different levels of the coagulation pathways. To address the ineffectiveness of heparin, exploring the biodiversity of the marine world can offer promising opportunities for the research and discovery of bioactive molecules with still little-exploited potential. As such, marine glycosaminoglycans (GAGs) are widely used as anticoagulants and anti-thrombotic agents. Indeed, new GAGs of marine origin have been extracted from marine invertebrates (sea cucumber, sea hare, ascidia, etc.) as well as from marine vertebrates (eel, corb, rabbit fish, smooth emissole, skate, etc.). This alternative to the use of marine glycosaminoglycans has several advantages. The first is the potential of using these biomolecules as alternatives to traditional heparin therapy without causing undesirable effects. The second is the possibility of eliminating the major risks of contamination due to unconventional pathogens (viruses, prions).

GAGs are anionic polymers of repetitive disaccharidic units. These units consist of uronic acid (AU) (D-glucuronic acid (GlcUA) or L-iduronic acid (IdoUA)) bound by β 1-3 or β 1-4 glycosidic bonds to hexosamine (N-acetyl-D-glucosamine (GlcNAc) or N-acetyl-D-galactosamine (GalNAc)) [4]. These polysaccharides are highly functional and are often extracted as a heterogeneous mixture. Among these GAGs are chondroitin sulfate and dermatan sulfate, which can have powerful anticoagulant effects and thus represent potential heparin-like alternatives of marine origin.

GAGs can interact with various biological systems such as wound regeneration [5], blood coagulation [6], thrombosis [7], cancer [8], inflammation [9], and retinal neovascularization [10].

In light of this, the goal of this work was to extract glycosaminoglycans with biological activities from fish by-products. The first step involved breaking down garfish by-product (Belone belone) into its constituent parts. This process involved chemical and structural analysis of the resulting materials. This work’s main objective was to isolate GAGs through enzymatic digestion, followed by cationic detergent purification and ethanol precipitation. The GAGs were then purified via DEAE-cellulose chromatography and lyophilized prior to being concentrated. After purification, the GAGs were analyzed through various post-processing techniques such as Fourier transform infrared spectroscopy, differential calorimetry, and SAX-HPLC. Additionally, this process also revealed the anticoagulant properties of these compounds.

## 2. Materials and Methods

### 2.1. Reagents

The chemical compounds and solvents used in this study were of analytical grade and used as-obtained from commercial sources. Proteases from *Bacillus licheniformis* (Alcalase^®^ 2.4L) was obtained from Novozymes^®^ (Copenhagen, Denmark). General GAGs, chondroitine sulfate from bovine trachea, dermatan sulfate from porcine intestinal mucosa, heparan sulfate from bovine kidney, and chondroitinase ABC (chondroitin ABC lyase) were purchased from Sigma–Aldrich (St. Louis, MO, USA). Unsaturated chondro/dermato disaccharides were obtained from Seikagaku Corporation (Tokyo City, Japan). Diethylaminoethyl-cellulose was obtained from Pharmacia (Uppsala, Sweden). All other reagents were of analytical grade.

### 2.2. Material Preparation

Garfish by-products (skin, heads, and bones) were recovered from the local Sfax market. Biological materials were transported from cold storage to the lab. Samples of garfish were washed two times in order to remove impurities and were then divided. Until analysis and use, samples were put into sealed plastic bags and stored at −20 °C.

### 2.3. Enzymatic Extraction of GAGs from Garfish By-products

GAGs were extracted according to the method outlined by Ben Mansour et al. [11]. First, 5 g of the ground material (heads, skin, and bones) was homogenized in 250 mL of sodium acetate buffer (0.1 M, pH 8). Thereafter, an enzymatic hydrolysis step was performed with Alcalase^®^ for 24 h at 50 °C. After the enzymatic hydrolysis, the mixture was heated for 20 min at 80 °C in order to inactivate the endogenous enzymes. The mixture was then left to cool down at room temperature and filtered. The residue was washed with distilled water and filtered again. The filtrates were mixed, and GAGs were precipitated with cetylpyridinium chloride 1% (*w*/*v*). After 24 h of incubation at room temperature, complexed glycosaminoglycans were recovered by centrifugation at 5000× *g* for 30 min at 4 °C. The pellets were solubilized in a minimum volume of NaCl/ethanol solution (100:15 *v*/*v*), and the mixtures were incubated for 24 h under agitation at 4 °C. Dissolved glycosaminoglycans were subsequently precipitated twice with absolute ethanol. The mixtures were incubated for 24 h at 4 °C and then centrifuged at 5000× *g* for 30 min. Finally, the collected GAGs were solubilized in a minimum amount of deionized water and lyophilized.

### 2.4. Purification of GAGs from Garfish by-products

Diethylaminoethyl-cellulose (DEAE-cellulose) allowed us to maintain an acidic pH and then separate the different component fractions of the GAGs according to their charges. Lyophilized GAGs were dissolved in distilled water and then deposited into a DEAE-cellulose column (2 cm × 6 cm) that was previously balanced with a 50 mM NaCl solution. The samples that were fixed in the column were eluted with a 50 mM NaCl solution and then with a 2.5 M NaCl solution. Then, three volumes of ethanol were added to the recovered fraction. The GAGs were precipitated at −20 °C for 24 h and then centrifuged at 10,000× *g* for 10 min. The collected pellets were freeze-dried and then stored at −20 °C for further use.

### 2.5. Glycosaminoglycans Yield Calculation

The obtained yields of the three GAGs from the skin, heads, and bones of garfish were calculated as follows:

Yield (%) = (weight of dried GAGs (g)/weight of dry raw material (g)) × 100

### 2.6. Chemical Composition Analysis

The amount of total sugars was measured according to the method of DuBois et al. [12] using phenol-sulfuric acid and using glucose as a standard. The toal uronic acid content was determined according to the method of Cesaretti et al. [13] using glucuronic acid as a standard. The sulfate content was assessed by the BaCl2/gelatin turbidimetric method [14].

### 2.7. Cellulose Acetate Electrphoresis Analysis

The purified GAGs acquired from the skin, bones, and heads of garfish were examined for the presence of different complexes via cellulose acetate electrophoresis that was executed in barium acetate [15]. A GAG standard mixture (35 µL) containing heparan sulfate, dermatan sulfate, and chondroitin sulfate (1 mg/mL) and 35 µL of the purified GAGs from the skin, heads, and bones of garfish (1 mg/mL) were placed at the origin of a cellulose acetate strip (Sartorius). Then, electrophoresis was carried out in a barium acetate buffer (0.1 M), which was run for two hours at 60 V. The cellulose acetate strip was then stained with toluidine blue. Thereafter, the GAG constituents were identified according to their electrophoretic mobility versus the respective components of the GAG standard mixture.

### 2.8. Visible Ultraviolet Spectrum of GAGs

The ultraviolet–visible absorption spectrum of the purified GAG solutions (1 mg/mL) was assessed with a UV–Vis spectrophotometer (JENWAY/7315, Staffordshire, UK) with a spectral range from 198 to 1000 nm and a quartz cuvette with a path length of 10 mm. Distilled water was used as a control.

### 2.9. Molecular Mass Analysis

The molecular mass of the three GAGs was determined by PAGE as described by Edens et al. [16]. The purified GAGs (15 mg) were layered on the gel and run at 100 V for 40 min. The gel was then stained with toluidine blue (0.1% in 1% acetic acid) for 30 min, followed by destaining in 1% acetic acid. The related calibration curve was constructed using oligosaccharide standards of known molecular mass prepared from CS. The molecular mass evaluation was performed via the densitometric acquisition of the bands and the comparison of their migration times on the calibration curve constructed by plotting the retention times of the standards against the logarithms of their molecular mass values.

### 2.10. Disaccharide Composition Determination

The recovered GAGs (50 μg) were homogenized with Tris-acetate buffer (50 mM, pH 8.0) and then incubated with chondroitin ABC lyase (0.5 U/mg sample) at 37 °C for 24 h. The obtained unsaturated disaccharides were quantified by strong anion exchange (SAX) by means of high-pressure liquid chromatography (SAX-HPLC) equipment equipped with a 150 mm × 4.6 mm spherisorb 5-SAX stainless-steel column (5 μm, trimethylammoniopropyl groups Si-CH_2_-CH_2_-CH_2_-N^+^ (CH_3_)_3_ in Cl^−^ form, from Phase Separations Limited, Deeside Industrial Park, Deeside Clwyd, UK), and detection was carried out at 232 nm. Isocratic separation was assessed at a flow rate of 1.2 mL/min using 50 mM NaCl (pH 4) for 5 min followed by a linear gradient from 5 to 25 min from 50 mM NaCl to 1.2 M NaCl (pH 4). Authentic unsaturated standard disaccharides were used for qualitative and quantitative purposes.

### 2.11. In Vitro Anticoagulant Activity

The impact of GAGs on the hemostatic system was evaluated by measuring the activated partial thromboplastin time (aPTT), prothrombin time (PT), and thrombin time (TT) using a semi-automatic line STA (Diagnostica Stago, Asnières sur Seine, France). Each of the three samples, GSB, GCB, and GHB, were dissolved in physiological serum. All analyses were performed in triplicate.

#### 2.11.1. Blood Collection and Preparation of PPP and PRP

Blood was obtained from healthy human volunteers exempt from medication for at least 10 days. All volunteers involved this study provided informed consent. Blood was collected via venipuncture into siliconized VacutainerTM tubes (Becton Dickinson, Le Pont de Claix, France) containing buffered sodium citrate (9:1, *v*/*v*) and centrifuged for 10 min at 200× *g* to obtain platelet-rich plasma (PRP). Platelet-poor plasma (PPP) was obtained by centrifugation of the remaining blood at 1200× *g* for 10 min.

#### 2.11.2. Activated Partial Thromboplastin Time Assay

The aPTT assay was assessed by mixing 5 µL of the purified GAGs (at different concentrations) with 45 µL of PPP and incubating the mixture for 3 min at 37 °C. Afterward, 50 mL of aPTT reagent (CK-PREST) was added, and the mixture was incubated for 3 min at 37 °C. The clotting time was immediately measured after the addition of 100 µL of 25 mM CaCl_2_. The clotting time was expressed in seconds and as a ratio, with the average value of a normal subject being less than 1.2. The enzyme activity control was measured by replacing the GAGs with physiological serum.

#### 2.11.3. Thrombin Time Assay

The TT assay was performed by mixing 10 µL of the purified GAGs (at different concentrations) with 90 µL of PPP. The mixture was incubated for 3 min at 37 °C. The clotting time was determined after the addition of 100 µL of thrombin (80 NIH). The TT value was expressed in seconds.

#### 2.11.4. Prothrombin Time Assay

For the PT assay, 5 µL of the purified GAGs (at different concentrations) was mixed with 45 µL of PPP and then incubated for 3 min at 37 °C. The clotting time was determined after the addition of 100 µL of Neoplastine^®^ CI (Diagnostica Stago). The PT value was expressed in seconds.

### 2.12. Statistical Analysis

All measurements were performed in triplicate, and all data are presented as means ± SD (*n* = 3). Mean significant differences were analyzed by Fisher’s test (ANOVA) using the SPSS software package (SPSS, Chicago, IL, USA).

## 3. Results

### 3.1. Extraction, Purification, and Chemical Composition of GAGs

**Figure 1:**
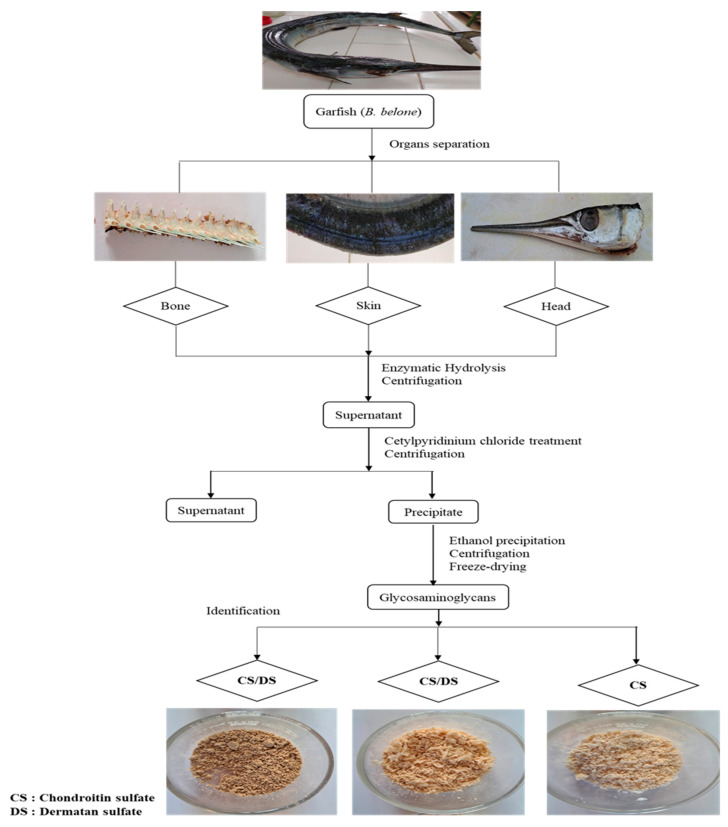
Extraction procedure of GAGs isolated from skin (GSB), bones (GCB), and heads (GHB) of garfish.

GAGs are a charged heterogeneous group of natural polysaccharides that are present in a wide diversity of biological entities, spanning internal compartments, cellular surfaces, and the external habitat. In the present work, GAGs were prepared from skin (GSB), bones (GCB), and heads (GHB) of garfish (*Belone belone*) through enzymatic breakdown and reseparation using DEAE-cellulose column chromatography (Figure 1). The existing techniques for extracting glycosaminoglycans depend on procedures that include tissue hydrolysis, protein elimination, and subsequent purification stages [17]. This method was optimized to yield exceptionally pure substances with a consistent composition and physicochemical characteristics [18].

The assessment of the physicochemical characterization and the resulting yield of GAGs prepared from different fish parts are listed in Table 1. The yields (based on the dry weight) obtained revealed that the heads (8.1%) were the best substrates followed by the skin (3.7%) and bones (1.4%). These variations in yields can result from a variety of factors such as fish species, sample preparation conditions, and extraction techniques [19]. For comparative purposes, the obtained yields of GAGs that were recovered from the heads and bones of *S. canicula* were around 6% and 2%, respectively [20].

The carbohydrate, uronic acid, and sulfate contents are also compiled in Table 1. Carbohydrates were the most abundant part in the GSB, GCB, and GHB (69 ± 4%, 70 ± 8%, and 70 ± 7%, respectively). The obtained results showed no significant differences (*p* > 0.05) between the GAGs extracted from the different fish parts. The uronic acid contents that were determined by means of the carbazole test were around 37 ± 3.5%, 35 ± 4%, and 35 ± 4.5% for the GSB, GCB, and GHB, respectively. Previously, GAGs have exhibited uronic acid percentages of around 23.9% and 41.2% for backwater clam [21] and shark [22], respectively. The sulfate contents of the GSB, GCB, and GHB were determined, reaching values of 20 ± 1%, 20 ± 1.5%, and 21 ± 2%, respectively. The considerable sulfation patterns indicated a strongly polyanionic structure for the GAGs derived from the different fish parts. The values recorded for the sulfate amounts were greater than those previously reported for GAGs extracted from *Sargassum tenerrimum* (6.60 ± 1.42%) [23], which was quite analogous to that acquired for GAGs obtained from mackerel fish waste (21%) [24]. Several investigations have observed unique sulfation profiles in fish cartilage as opposed to those seen in terrestrial vertebrates [25]. Conversely, Duarte et al. [26] proposed that the physiological impacts of polysaccharides are associated with the extent and positioning of sulfation.

Testing of the homogeneity of the variance was performed to justify the employment of the ANOVA test. The test indicated that there were no significant differences between the variances for the three studied compounds (sulfate, uronic acid, and total sugar). In fact, their respective *p*-values (0.501, 0.945, and 0.613) were higher than the 5% probability level, which indicated that the variances were homogeneous and justified the application of the ANOVA test (Table 2).

### 3.2. Purity Identifcation and Cellulose Acetate Electrophoresis Analysis

**Figure 2:**
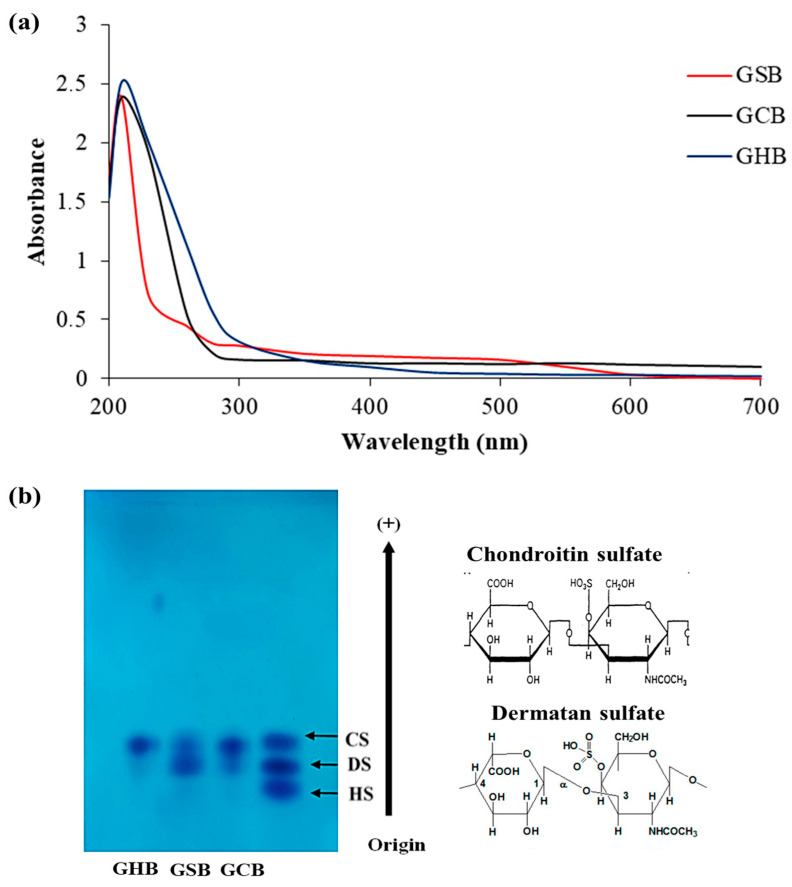
(**a**) UV spectrum of GAGs from skin (GSB), bones (GCB), and heads (GHB) of garfish in the wavelength range from 190 to 700 nm obtained using a UV–vis spectrophotometer. (**b**) Acetate cellulose electrophoresis of GSB, GCB, and GHB. Standard GAGs: dermatan sulfate (DS), chondroitin sulfate (CS), and heparan sulfate (HS).

The purified GAGs were analyzed using UV–visible spectroscopy across the wavelength spectrum from 190 to 700 nm (Figure 2a). The UV absorption curves of the GSB, GCB, and GHB mostly overlapped, and they exhibited a maximum absorption at 210 nm, as is characteristic of polysaccharides [27]. The weak absorption peaks observed within the GSB spectrum, notably at 260 nm, signified the existence of small concentrations of nucleic acids. The lack of any substantial absorption at 280 nm confirmed the limited protein content in the samples [28].

Cellulose acetate electrophoresis has found extensive application in the fractionation of combined GAGs and in evaluating their degrees of purity. As shown in Figure 2b, the analysis of the GSB and GCB by cellulose acetate electrophoresis showed two different bands. The initial group migrated as far as CS, whereas the second group was recognized as DS. Conversely, the cellulose acetate electrophoresis analysis for the GHB indicated the existence of a single band that corresponded to CS. CS/DS as a copolymeric structure was reported earlier in many fish species, and its structural variability is known to be tissue-dependent [29]. The existence of disaccharides containing iduronic acid imparts an inherent flexibility to the polymer, allowing it to interact with other molecules more effectively. In the study of Zhang et al. [30], the analysis of GAGs from red salmon heads by agarose gel electrophoresis indicated the presence of CS as a principal GAG. Furthermore, the cellulose acetate analysis of GAGs derived from *Scyliorhinus canicula* by-products revealed the presence of both CS and DS [31]. Previous investigations have shown differences in the relative amounts, varieties, and molecular sizes of CS and DS among GAG samples collected from diverse fish species and tissues [32]. However, the same tissue from different fish had, in general, the same types of GAGs but with different chemical compositions.

### 3.3. Molecular Weight Analysis of GAGs

**Figure 3:**
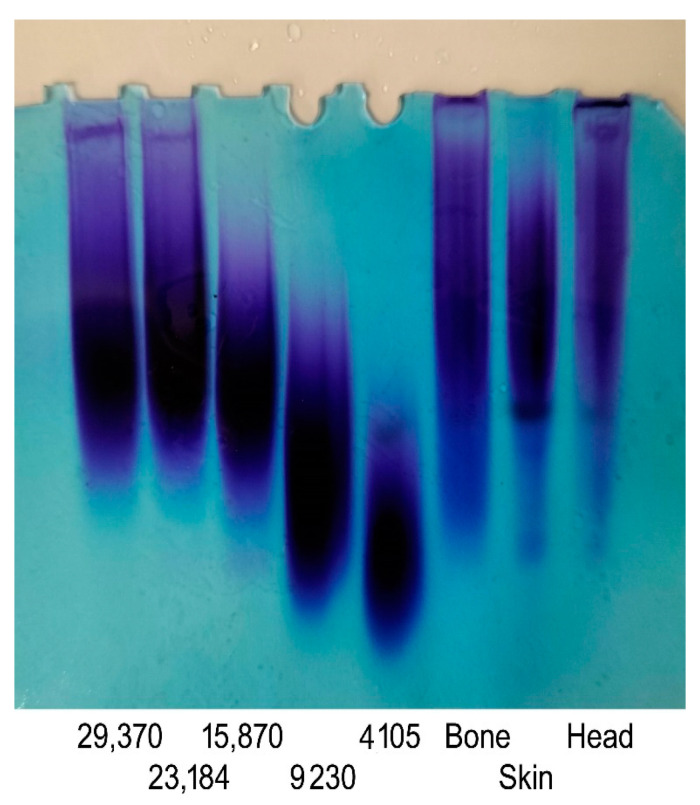
PAGE analysis of GSB, GCB, and GHB. The calibration curve was determined using CS standards with known molecular mass and with masses of 29,370, 23,184, 15,870, 9230, and 4105 Da.

GAGs obtained from different origins exhibit diversity in terms of their molecular weight, structures, and characteristics. The molecular weight of GAGs plays a crucial role in shaping their functional attributes and their impact on biological activities [33]. In the current work, molecular weight measurements of the GAGs were performed by gradient PAGE using a series of standard CS with a known molecular weight. As shown in Figure 3, the PAGE analysis of the GAGs revealed a single symmetrical peak consistent with a relatively homogenous polysaccharide preparation. This result confirmed the high purity of the obtained GAGs, as suggested by the cellulose acetate electrophoresis results. Based on calibration with standards, the molecular weights of the GSB, GCB, and GHB were estimated to be 37.85 kDa, 34.41 kDa, and 46.13 kDa, respectively (Table 2). Interestingly, the GAGs from the skin and bones showed very similar properties, making these samples an adequate source of CS/DS with similar molecular weights. Sources in the literature have documented variances in the molecular weights of CS and DS samples obtained from various fish species. For instance, CS derived from the heads of silver-banded whiting fish reportedly exhibited a molecular weight of 64 kDa, while CS purified from bony fish ranged from 37–40 kDa in weight [25], and CS/DS derived from corb bones exhibited a lower molecular weight of 23.35 kDa. According to previous studies, CS derived from cartilaginous fishes generally shows molecular weight values between 50 and 70 kDa. On the contrary, CS purified from terrestrial sources has a lower molecular weight that ranges between 13 and 26 kDa [19].

### 3.4. Disaccharide Composition Analysis

**Figure 4:**
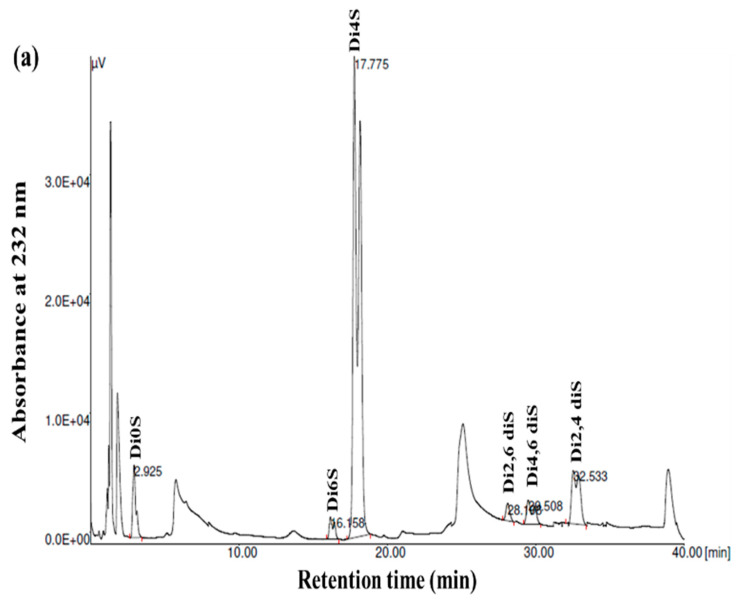

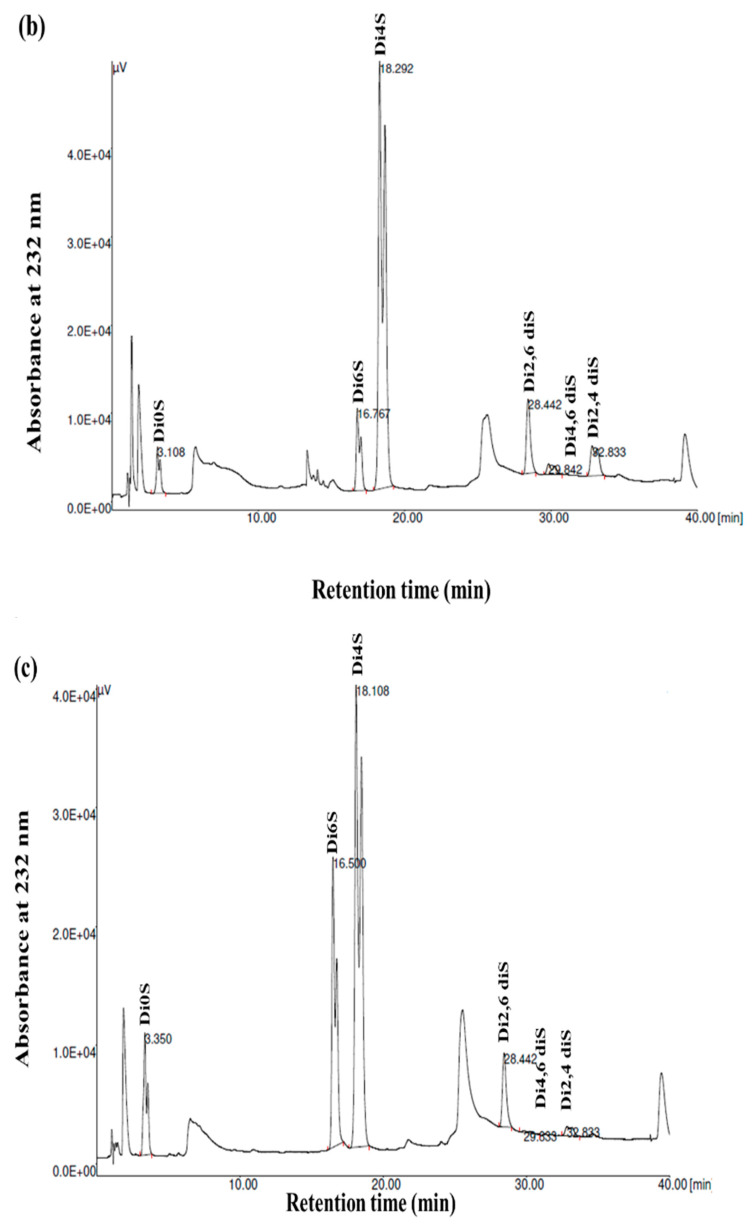
SAX-HPLC separation of the unsaturated disaccharides produced from GSB (**a**), GCB (**b**), and GHB (**c**) of garfish and treated with chondroitinase ABC. ΔDi0S (ΔUA-GalNAc), ΔDi6S (ΔUA-GalNAc 6S), ΔDi4S (ΔUA-GalNAc 4S), ΔDi2, 6diS (ΔUA2S-GalNAc 6S) ΔDi4, 6diS (ΔUA GalNAc4, 6diS), ΔDi2, 4diS (ΔUA2S-GalNAc4S). The disaccharide species were identified by coelution with purified standards (Seikagaku Corporation/Sigma–Aldrich).

GAGs are generally heterogeneous in terms of their main structural features, including their sulfation pattern, molecular weight, composition, and charge density. To acquire more comprehensive data regarding the disaccharide composition of the samples, the GSB, GCB, and GHB were digested with chondroitinase ABC, which was followed by an analysis of the composition of the unsaturated disaccharides using SAX-HPLC (Figure 4). Chondroitinase ABC is a non-specific lyase that acts on both CS and DS. This enzyme successfully generated three unsaturated disaccharides: non-sulfated ΔDi0s, singly sulfated ΔDi6S, and ΔDi4S. Subsequently, a chemical composition analysis was undertaken to reveal the structural distinctions among these three types of GAGs.

As shown in Figure 4 and Table 3, the chondroitinase ABC produced different unsaturated disaccharides in various amounts. In fact, the three GAGs were mainly composed of similar unsaturated disaccharides; however, there was no single tendency in terms of the percentages. Specifically, a minimal amount of non-sulfated disaccharides was revealed by SAX-HPLC (4–9%) in the three GAGs. These results were in a similar range (1.6–12.9%) to those described for different marine species [25]. In addition, the levels of monosulfated disaccharide, ΔDi4S, were evaluated to be 74.78%, 69.22%, and 57.17% for the GSB, GCB, and GHB, respectively. These compounds were also determined to consist of monosulfated disaccharide, ΔDi6S, ranging from 2.57% to 25.55%. The results revealed a greater presence of sulfate groups at position 4 as opposed to position 6, with 4S/6S ratios that were lower for the GHB and GCB (2.23 and 7.40, respectively) compared to the GSB (29.09). Large-scale manufacturing of chondroitin sulfate (CS) primarily originates from shark cartilage, which is rich in ΔDi-6S (68%) and ΔDi-2S,6S (11%) [34]. Previously, CS derived from codfish bones was reported to be composed of 7.09% ΔDi0S, 73.85% ΔDi4S, and 19.06% ΔDi6S [28]. Moreover, disulfated disaccharides were observed in all GAGs, even if in different percentages. Interestingly, the disulfated species, ΔDi2,6diS, was present in the obtained GAGs in a range of 1.71–6.28%, while the disulfated disaccharide ΔDi2,4diS was found in an elevated concentration only in the GSB (10.92%). The disulfated disaccharide ΔDi4,6diS of the GSB, GCB, and GHB accounted for approximately 4.14%, 2.05%, and 0.37%, respectively. The existence of disulfated disaccharides resulted in a notable elevation in the overall charge density, ranging from 0.99 to 1.12. This measurement was generally higher than that of mammalian CS [35]. These results have shown that GSB and GCB are sulfated, particularly in positions 4 (ΔDi4S) and positions 2 and 4 (ΔDi 2,4diS), confirming that these polymers mainly contained GlcA/IdoUA as uronic acid [36]. These findings were also in accordance with the cellulose acetate electrophoresis results of the samples. Marine fish have traditionally been used as the primary resource for extracting CS and DS; nevertheless, their structures differ across different species [29]. Moreover, CS derived from rabbit fish heads was shown to be composed of 17.21% ΔDi2,6diS, 2.6% of ΔDi4,6-diS, and 1.03% ΔDi2,4diS [37].

### 3.5. In Vitro Anticoagulant Activity of Purified GAGs

Anticoagulants are widely employed in the healthcare sector to treat critical conditions like strokes and heart attacks, both of which contribute significantly to mortality rates. Heparin has been widely utilized as an anticoagulant for numerous years. While heparin presents benefits in handling thromboembolic diseases, its application is limited due to possible health risks for both humans and animals. Over the past few years, there has been an increasing commitment to investigating alternatives to heparin [9,11]. It is now acknowledged that specific polysaccharides can exhibit biological effects, including GAGs, which are frequently used as anticoagulant agents. These polysaccharides have been demonstrated to display anticoagulant characteristics throughout various phases of the coagulation process. They accomplish this by blocking the initial response of the extrinsic pathway and also by suppressing tenase and thrombin.

The assessment of anticoagulant efficacy is typically determined through traditional coagulation assessments such as the activated partial thromboplastin time (aPTT), prothrombin time (PT), and thrombin time (TT) [38]. Specifically, the aPTT and PT are linked to the intrinsic and extrinsic coagulation pathways, respectively, while the TT is utilized as an indicator for the quantity and coagulation effectiveness of fibrinogen within the plasma during the final stages of the coagulation process. Many reports have suggested that the activated partial thromboplastin time test provides valuable information regarding the risk during evaluations, the monitoring of anticoagulant therapy with heparin, and the evaluation of factors linked to the risk of thrombosis.

**Figure 5:**
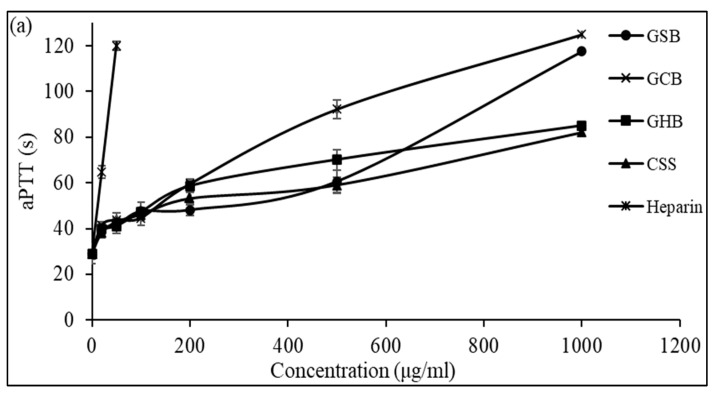

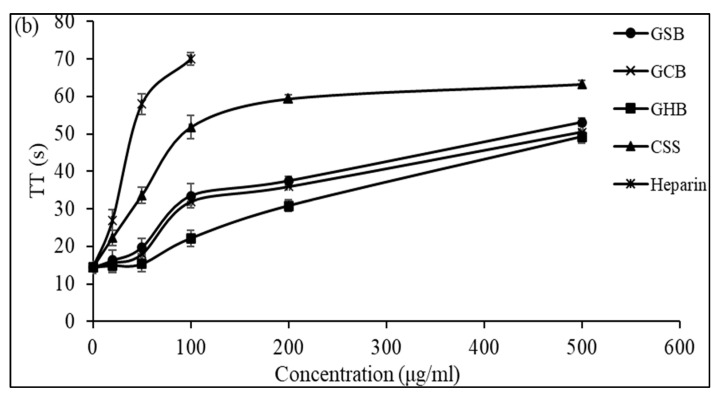
Anticoagulant activity of GSB, GCB, and GHB at different concentrations evaluated by the measurement of (**a**) the activated partial thromboplastin time (aPTT) and (**b**) thrombin time (TT). Heparin and chondroitin sulfate (CSS) were used as references.

The anticoagulant activity of the GSB, GCB, and GHB determined by the aPTT test is shown in Figure 5a. The results showed that the three GAGs could prolong the aPTT in a dose-dependent manner. At a concentration of 1000 μg/mL, the GHB and GSB caused a strong prolongation of the coagulation times, which were about 2.93 and 4.05 times greater than that of the negative control, respectively. However, at a concentration of 500 μg/mL, the aPTT determined in the presence of GCB was prolonged by 3.18 times compared to that of the control plasma. The potent effects of the three GAGs determined by the aPTT test suggested an inhibition of the intrinsic and common coagulation pathways. In a similar context, Dhahri and colleagues (reference [39]) documented that a sulfated polysaccharide extracted from the viscera of *Bursatella leachii* extended the thrombin time (TT) by a factor of two when exposed to 5 μg/mL and significantly increased the activated partial thromboplastin time (aPTT) by a factor of 4.5 with the introduction of 25 μg/mL.

Additionally, Ben Mansour et al. [11] illustrated concentration-related anticoagulant effects, as assessed by aPTT, which were two to three times less potent compared to heparin when applied to the skin of a ray (*Raja radula*). All the GAGs contained non-sulfated disaccharide ΔDi-0S, mono-sulfated disaccharides ΔDi-6S and ΔDi-4S, and di-sulfated disaccharide ΔDi2,6-diS. This implies that the disaccharide composition, especially the number and location of sulfate units, might play a crucial role in the biological activity of GAGs. These results might explain why the GCB and GSB exhibited good anticoagulant activity, as they were composed of a high amount of ΔDi-4S and ΔDi2,4-diS. The thrombin time (TT) reflects the coagulation system’s pathway. We assessed the anticoagulant properties of the three GAGs by measuring their effect on the plasma clotting time using a TT assay and comparing the results to a negative control (Figure 5b). With increasing concentrations of GSB, GCB and GHB, the anticoagulant activity increased. Interestingly, the GSB, GCB and GHB prolonged the TT by about 3.78, 3.56, and 3.41 times, respectively, compared to that of the control at a concentration of 500 μg/mL. The elongation of the TT suggests the suppression of thrombin activity or fibrin formation, a process reliant on thrombin inhibition and that impacts the coagulation duration. In addition, these polymers prolonged the TT more than that previously described for CS from shark cartilage (1.3-fold at a concentration of 1000 μg/mL) [40]. Hence, the anticoagulant properties of the three GAGs can be ascribed to their capacity to impede the generation of fibrin, which is connected to the suppression of the intrinsic pathway.

A PT assay (which evaluates the extrinsic pathway of coagulation) was also carried out to assess the anticoagulant activity of the three GAGs. The obtained results showed that no enhancement of the PT activity was observed as it remained unchanged even at the highest GAG concentration used (1000 μg/mL), and this indicated that these GAGs did not have any effect on the extrinsic coagulation pathway. This was consistent with previously reported results of GAGs in terms of their anticoagulant activity [38]. According to Ben Mansour et al. [11], sulfated glycosaminoglycans from different marine species exhibited anticoagulant effects in aPTT and TT assays, but not in a PT assay. Research has established a connection between the coagulation properties and the structural attributes of GAGs, such as the level of sulfation, sulfation arrangements, molecular mass, sugar composition, glycosidic branching, and the three-dimensional arrangement of the sulfated polysaccharide. In this context, Yuan et al. [41] established that a monosulfated disaccharide at position 4 (CS-A) and/or a disulfated disaccharide at positions 2 and 4 (CS-B) are required for the inhibition of thrombin. This inhibition occurs by stimulating heparin cofactor II and antithrombin, respectively. In another study, Liang et al. [42] reported that an increase in the DS content and molecular weight generally corresponds to an increase in the anticoagulant activity of GAGs. Moreover, Pavao et al. [43] reported that 4-O-sulfation of the N-acetyl-b-D-galactosamine is essential for the anticoagulant activity of DS. In summary, increasing the DS and molecular weight of glycosaminoglycans generally enhances their anticoagulant activity. However, the specific effects may depend on the type of GAG and its sulfation pattern. This is in line with the results of our SAX-HPLC analysis. The results obtained from the aPTT, TT, and PT tests suggest that the three glycosaminoglycans (GAGs) have the ability to influence the intrinsic blood clotting factors and manage the degree of fibrinogen conversion into fibrin by modulating thrombin function.

## 4. Conclusions

In summary, the constitution and configuration of sulfated GAGs extracted from garfish (*Belone belone*) skin (GSB), bones (GCB), and heads (GHB) were extensively scrutinized through cellulose acetate electrophoresis, PAGE analysis, and disaccharide composition analysis. The PAGE analysis showed that the GSB, GCB, and GHB had relatively high molecular weights of 37.85 kDa, 34.41 kDa, and 46.13 kDa, respectively. The examination of the purified CS/DS using SAX-HPLC after treatment with specialized chondroitinases showed that these polymers were composed of different ratios of unsulfated disaccharides, singly sulfated disaccharides, and doubly sulfated disaccharides. In addition, the CS/DS showed potent anticoagulant activities through measurements of the activated partial thromboplastin time and thrombin time. Based on the data collected, it is logical to infer that CS/DS extracted from discarded garfish components could find practical use in scientific and pharmacological contexts.

## Figures and Tables

**Table 1:** Chemical composition (calculated based on dry matter) of GAGs from skin (GSB), bones (GCB), and heads (GHB) of garfish. Values are given as mean ± SD from triplicate determinations.

Composition (%)	GAGs	Mean ± SD
Yield	GSB	3.7
	GCB	1.4
	GHB	8.1
Sulfate	GSB	20 ± 1
	GCB	20 ± 1.5
	GHB	21 ± 2
Uronic acid	GSB	37 ± 3.5
	GCB	35 ± 4
	GHB	35 ± 4.5
Total sugar	GSB	69 ± 4
	GCB	70 ± 8
	GHB	70 ± 7

**Table 2:** Analysis of variance for the impact of GAGs source.

		Sum of Squares	dF *	Mean of Squares	F Test	*p*-Value
Sulfate	Inter-groups	0.249	2	0.124	0.049	0.952
	Intra-groups	15.201	6	2.533		
	Total	15.449	8			
Uronic Acid	Inter-groups	7.362	2	3.681	0.225	0.805
	Intra-groups	98.286	6	16.381		
	Total	105.648	8			
Total Sugar	Inter-groups	4.389	2	2.194	0.048	0.954
	Intra-groups	274.333	6	45.722		
	Total	278.722	8			

Results are the average of three independent determinations ± SD. * dF: degrees of freedom.

**Table 3:** Amount, disaccharide composition, and charge density values of GAGs from garfish by-products. Values are given as mean ± SD obtained from triplicate determinations. ΔUA, 4,5-unsaturated uronic acid; GalNAc, N-acetyl-galactosamine; S, sulfate group. The percentage of each identified disaccharide was determined by purified standards (Seikagaku Corporation/Sigma–Aldrich) and reported as weight percent. Charge density was calculated by considering the mean of sulfate group per disaccharide unit.

Disaccharide Composition (%)	GSB	GCB	GHB
ΔDi0S (ΔUA-GalNAc)	5.87	4.44	9.03
ΔDi6S (ΔUA-GalNAc 6S)	2.57	9.35	25.55
ΔDi4S (ΔUA-GalNAc 4S)	74.78	69.22	57.17
ΔDi2, 6S (ΔUA2S-GalNAc 6S)	1.71	8.37	6.28
ΔDi4, 6S (ΔUA 4S-GalNAc 6S)	4.14	2.05	0.37
ΔDi2, 4S (ΔUA2S-GalNAc 4S)	10.92	6.55	1.57
Charge density	1.11	1.12	0.99
4S/6S ratio	29.09	7.40	2.23
Molecular weight (kDa)	37.85	34.41	46.13

## Data Availability

The data presented in this study are available on request from the corresponding author.
